# Evolving CAR-T-Cell Therapy for Cancer Treatment: From Scientific Discovery to Cures

**DOI:** 10.3390/cancers16010039

**Published:** 2023-12-20

**Authors:** Avisek Majumder

**Affiliations:** Department of Medicine, University of California San Francisco, San Francisco, CA 94158, USA; avisek.majumder@ucsf.edu

**Keywords:** immunotherapy, immuno-oncology, combination therapy, cancer treatment, tumor microenvironment (TME), chimeric antigen receptor (CAR), checkpoint blockade, immunomodulation, solid tumor, hematological malignancies, blood cancer, B-cell lymphoma, cytokine release syndrome, TCR-T-cell receptor

## Abstract

**Simple Summary:**

It is well recognized now that the development of drug resistance is one of the leading causes of treatment failure in conventional therapies. In comparison, recent improvements in immunotherapy showed promising results in eradicating cancer. Chimeric antigen receptor (CAR)-T-cell therapy is one of the cancer immunotherapies that uses patient’s T cells and genetically modifies them to target cancer cells. Although CAR-T-cell therapy has shown remarkable success in treating blood cancer, it has proven far more limited in the treatment of solid tumors in different organs. This review article discussed the chronological development of CAR-T-cell therapies and their treatment options in different types of cancers. This article also addresses the current clinical challenges in CAR-T-cell therapy and recent advancements in developing novel therapeutic strategies with fewer side effects.

**Abstract:**

In recent years, chimeric antigen receptor (CAR)-T-cell therapy has emerged as the most promising immunotherapy for cancer that typically uses patients’ T cells and genetically engineered them to target cancer cells. Although recent improvements in CAR-T-cell therapy have shown remarkable success for treating hematological malignancies, the heterogeneity in tumor antigens and the immunosuppressive nature of the tumor microenvironment (TME) limits its efficacy in solid tumors. Despite the enormous efforts that have been made to make CAR-T-cell therapy more effective and have minimal side effects for treating hematological malignancies, more research needs to be conducted regarding its use in the clinic for treating various other types of cancer. The main concern for CAR-T-cell therapy is severe toxicities due to the cytokine release syndrome, whereas the other challenges are associated with complexity and immune-suppressing TME, tumor antigen heterogeneity, the difficulty of cell trafficking, CAR-T-cell exhaustion, and reduced cytotoxicity in the tumor site. This review discussed the latest discoveries in CAR-T-cell therapy strategies and combination therapies, as well as their effectiveness in different cancers. It also encompasses ongoing clinical trials; current challenges regarding the therapeutic use of CAR-T-cell therapy, especially for solid tumors; and evolving treatment strategies to improve the therapeutic application of CAR-T-cell therapy.

## 1. Introduction

**Figure 1:**
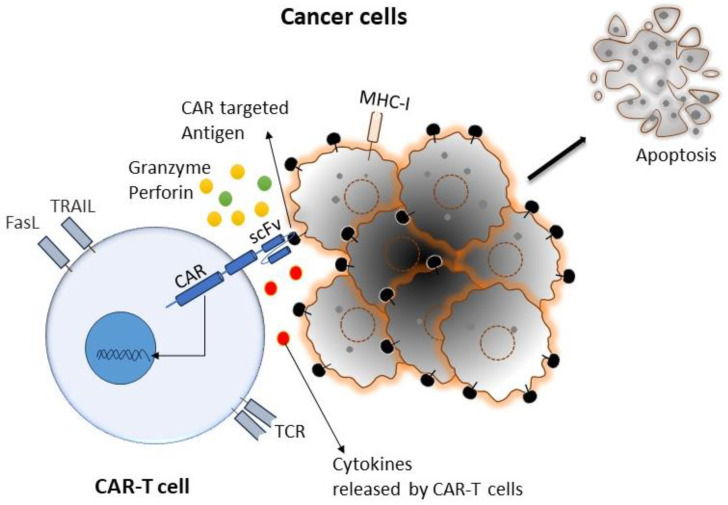
Cartoon diagram representing a typical interaction mechanism of CAR-T cells with targeted cancer cells: CAR-T cells can recognize tumor cells by binding with specific tumor-associated antigens (TAA) independent of TCR-MHC/peptide interactions. As a result, T cells are activated. CAR-T cells can induce apoptosis via the secretion of perforin, granzymes, and different pro-inflammatory cytokines, as well as the expression of the Fas ligand (FasL) and tumor necrosis factor-related apoptosis-inducing ligand (TRAIL).

Cancer is the primary health concern and leading cause of death worldwide. The American Cancer Society estimated that 1,958,310 new cancer cases and 609,820 cancer deaths occurred in the year 2022 in the United States [1]. Although there has been enormous progress in different types of conventional therapies for treating cancer, the development of drug resistance and drug-related toxicity makes these therapies much less effective. On the other hand, immunotherapy relies on using patients’ immune systems to identify cancer cells as foreign bodies and eradicate them via various mechanisms. Various immunotherapies have been developed for cancer, primarily by potentiating the body’s immune cells by releasing their immune suppression or empowering them to more effectively perform their immune functions. Chimeric antigen receptor (CAR)-T-cell therapy is one of the approaches of adoptive T-cell transfer (ACT) used in immunotherapy, which recently showed enormous success in terms of effectiveness and durable clinical response [2]. In this approach, T cells have been genetically altered by expressing the chimeric antigen receptor (CAR) to recognize tumor-specific antigens without the involvement of major histocompatibility complex (MHC), resulting in vigorous T-cell activation and robust anti-tumor responses [3]. CAR-T cells mediate anti-tumor effects through granzyme release, cytokine release, and other immune effectors (Figure 1).

Growing and evolving research has utilized sophisticated ex vivo culture and cellular engineering approaches to improve CAR-T-cell therapy, with long-life therapeutic responses. As a consequence of unparalleled clinical efficacy in particular B-cell malignancies, the US Food and Drug Administration (FDA) approved many CAR-T-cell therapies for treating hematological malignancy. CAR-T-cell therapy showed remarkable efficacy and profound therapeutic potencies in treating a subset of hematological malignancies by targeting lineage-restricted surface molecules [4,5,6,7,8,9]. In contrast, CAR-T-cell therapy hits roadblocks in treating solid tumors, mainly due to lack of high-quality antigen targets and poor tumor infiltration [10]. So far, most antigens targeted in solid tumors via CAR-T cells are mainly tumor-associated antigens rather than tumor-specific ones, which means that these antigens express higher levels in tumors and lower levels in some of the normal healthy tissues. These low antigen expression in normal tissue often leads to severe on-target/off-tumor toxicities. A case report showed severe side effects on a patient’s lungs after the infusion of CAR-T cells targeting HER2 antigen, which finally leads to death after a few days [11]. CAR-T cells’ clinical success also depends on CAR-T cells’ persistence; in this regard, CAR-T cells that exhibit a long-lived memory phenotype have shown much more antitumor responses than terminal effector-like CAR-T cells [12]. Antigen-independent tonic signaling is one of the main factors affecting CAR-T cells’ phenotype and longevity, there by its therapeutic response [13]. Many studies have tested innovative techniques to overcome these limitations, including targeting dual antigens, post-translational modified tumor-associated antigens, combination therapies to reduce immunosuppressive TME, and improved penetrations to the tumor site.

This article summarizes bench-to-bedside CAR-T-cell research, different clinical trials that have either been completed or are currently undergoing, limitations of this therapy, and different combinations of strategies to overcome the limitations of CAR-T-cell therapy. This review article will help future researchers and clinicians to understand the growing body of research and make newer therapeutic interventions that are more effective and have fewer side effects than current regimens of these patients.

## 2. Biology of CAR-T Cells in Cancer

**Figure 2:**
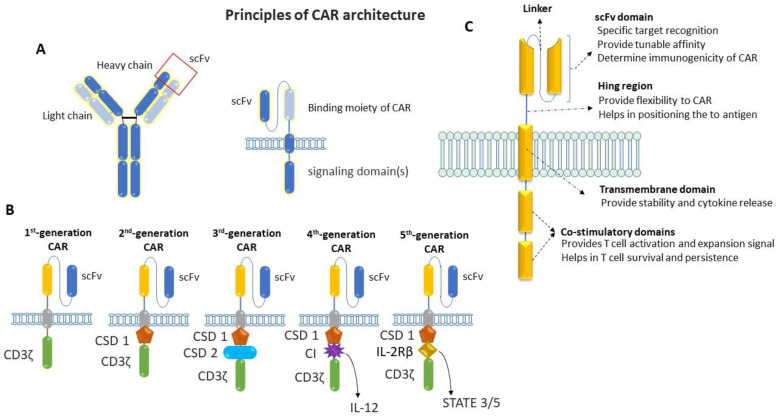
Schematic representation of the basic principle of CAR architecture. (**A**) Cartoon diagram showing the binding moiety of CAR, where scFv comes from a TAA-specific monoclonal antibody and the signaling domain(s) comes from activating and co-stimulatory immune receptors. (**B**) Cartoon diagram showing the progressive evolution of CAR-T cells from first generation to fifth generation: first-generation CAR contains a single-chain variable fragment (scFv) and CD3ζ for signal transduction; an additional co-stimulatory domain (co-stimulatory domain 1: CSD 1) was added on first-generation CAR to make a second generation, whereas two additional co-stimulatory domains (CSD1 and CSD 2) were added on first-generation CAR to make third-generation CAR; one additional domain for cytokine-expression (cytokine inducer: CI) was added on second-generation CAR to make fourth-generation CAR; fifth-generation CAR contains a co-stimulatory domain (co-stimulatory domain 1) and an additional domain that activates signaling pathways such as STATE3/5. (**C**) Cartoon diagram showing the basic structure of a typical CAR with the functions of individual domains.

Chimeric antigen receptors (CARs) are engineered to repurpose them to recognize specific antigens on the cancer cell’s surface. CAR contains a binding moiety in the extracellular part that recognizes and binds to target antigens in cancer cells and signaling domain(s) in the intracellular part that activates the T cells to proliferate and attack cancer cells (Figure 2). The binding moiety has a specific region called a single-chain variable fragment (scFv) that identifies specific antigens. This scFv region is derived from variable heavy (VH) and light (VL) chains of monoclonal antibodies connected via a flexible linker, as shown in Figure 2A. In comparison, the intracellular domain of CAR consists of several parts, which include a CD3ζ signaling domain (that activates the T cell) and co-stimulatory domains (that provide additional signals) [14,15,16,17,18]. Between the extra- and intra-cellular domains, there is a hinge region that provides flexibility to the extracellular domains (ECD) to help them to better recognize antigens and the transmembrane domain that anchors the CAR in the T-cell membrane.

The first generation of CAR mainly consists of scFv in the ECD and the CD3ζ chain of the T-cell receptor in the intracellular domain (Figure 2B). Although the first generation of CARs has been shown to activate T cells and induce cytokine release, they have less potency and persistency [14]. After that, the second generation of CAR was developed, with additional co-stimulatory domains to overcome these limitations of the first generations of CARs. One more co-stimulatory domain was added to the intracellular domain to develop the third generation of CARs [19,20]. This fourth generation of CARs is based on second-generation CARs, which contain an additional transgene for cytokine release. This fourth generation of CARs can activate T cells and release specific cytokines like IL-2 or IL-18, which is required to activate other immune cells and neutralize the immunosuppressive cytokines released by tumors [21,22,23]. After that, the fifth generation of CARs emerged to perform tasks such as targeting cancer cells, reducing inflammation, and recruiting other immune cells to fight cancer. In the fifth generation of CAR, a truncated intracellular domain of interleukin-2 receptor (IL-2R) was added, with a binding motif for transcription factors like STAT-3/5. In addition to activating CAR-T cells and promoting the generation of memory T-cells, the fifth generation of CARs reactivates and stimulates the immune system [24]. In addition to intracellular domains, other parts of this CAR (including the transmembrane domain and hinge region) have been modified for better recognition and binding with target antigen (Figure 2C) [25]. Due to the high antitumoral activity, potency, and persistence of the fifth generation of CARs, they have become a promising area of research in cancer immunology.

## 3. Generation of CAR-T Cells

**Figure 3:**
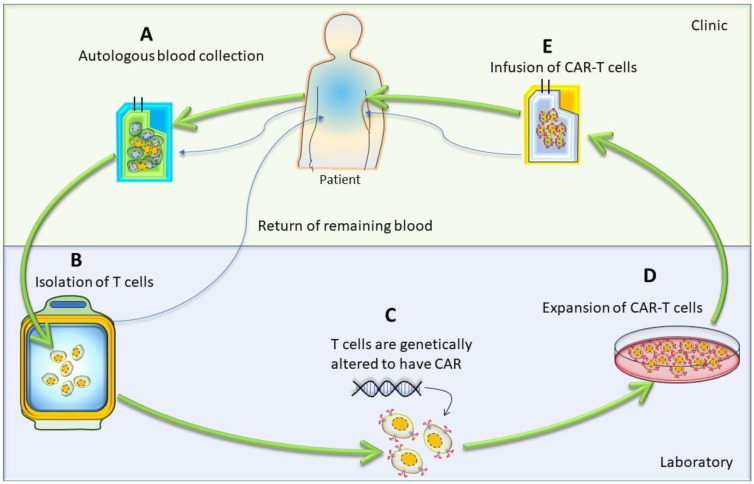
Cartoon diagram showing different stages of autologous CAR-T-cell production: The generation of autologous CAR-T cells started with the leukapheresis of a patient (**A**), followed by the separation of T cells (**B**); then, T cells were transduced to permanently integrate CAR transgene (**C**). After that, these genetically modified CAR-T cells expanded (**D**) and infused back into the patient (**E**).

The first step in CAR-T-cell therapy is to collect T cells from the patient (autologous) or a donor (allogenic). Then, these T cells need to be purified and genetically engineered to express artificially generated CARs, as shown in Figure 3 [26]. An engineered CAR gene is transduced in T cells via different methods, which include viral (lenti/retrovirus), non-viral (transposon or CRISPR/Cas9), and electroporation methods [27,28,29,30]. Next, to produce a large quantity of engineered CAR-T cells, these cells were expanded in vitro. After achieving the desired quantity and quality, they were infused back into the patient’s bloodstream. Generally, patients undergo chemotherapy before the infusion of CAR-T cells into the blood stream, which allow engineered T-cells to grow and kill cancer cells, as discussed in the Section 6.1 of this article. When CAR-T cells enter the patient’s bloodstream, they reach the cancer site, recognize specific antigens via CAR, and start performing their function, as detailed in the subsequent paragraphs and illustrated in Figure 1 [29].

## 4. CAR-T-Cell Therapy: From Scientific Discovery to Cures

Although CAR-T-cell therapy has recently gained much attention due to its revolutionary success in treating blood cancer, the idea has existed for several decades. It was repurposing the T-cell via genetically engineered T-cell receptors (TCRs) to kill cancer cells for the first time that was proposed in the 1980s. This approach had limitations because, at that time, it was challenging to identify specific antigens specific to cancer and make TCR specific to that antigen. This approach was even more challenging since TCR recognizes antigens in the major histocompatibility complex (MHC) setting, which may vary from patient to patient. To overcome these challenges, in 1989, it was proposed to combine the antigen-binding domain (derived from a monoclonal antibody) with the signaling domains of TCRs. This was the first generation of CAR-T cells; this approach allows the T cells to recognize the specific antigen present in cancer cells independently of MHC [31]. Although this first-generation CAR-T-cell therapy approach is more versatile than TCR, these cells have limited tumor-killing capacity because they do not grow and persist near tumors [32]. These limitations make the first generation of CAR-T cells less out of reach to the expectations of treating cancer. In the mid-1990s, the second generation of CAR-T cells was developed by adding the co-stimulatory domain to the CAR, which helps these cells to expand and survive. This approach has shown to be more efficient in preclinical models and paved the way for clinical trials. In 2002, the first effective CAR-T cells were developed by adding CD28 as a co-stimulatory domain [33]. In 2003, a study showed the complete abolition of B-cell lymphoma in a mouse model via CAR-T cells targeting the B-cell antigen CD19 [34]. In 2006, the first clinical trial was conducted with CAR targeting the tumor antigen carbonic anhydrase IX in patients with metastatic renal cell carcinoma [35]. Although this therapeutic strategy was reported to be safe for the patients, it did not yield any clinical benefits for the patients. After this step, in 2011, another clinical trial was conducted at the University of Pennsylvania, where they found clinical success in treating advanced chronic lymphocytic leukemia (CLL) using CAR-T-cell therapy [36]. This group used autologous T-cells engineered to express a CAR targeting the B-cell antigen CD19, and all patients were found to achieve complete remission after this therapy. This success led to many clinical trials with CAR-T-cell therapies, and they found various degrees of success. In 2013, a clinical trial with CAR-T-cell therapy found profound antitumor effects in patients with relapsed or refractory acute lymphoblastic leukemia (ALL) [37]. After that, in 2014, a group developed CAR-T-cell therapy for solid tumors to recognize antigen mesothelin [38]. Shortly after that, in 2015, the fourth generation of CAR-T cells was developed by adding a transgene with the second generation of CAR. 

After the CRISPR system was developed, this gene-editing tool was used to make CAR-T cells in 2017 [39]. Seeing the unprecedented success of CAR-T-cell therapy, in the year 2017, the US Food and Drug Administration (FDA) approved the CAR-T-cell therapy tisagenlecleucel (Kymriah) for the treatment of relapsed or refractory ALL [40]. Consequently, the FDA approved axicabtagene ciloleucel (Yescarta), another CAR-T-cell therapy for treating relapsed or refractory diffuse large B-cell lymphoma (DLBCL) in adults [40]. After that, one by one, several CAR-T-cell therapies were approved for treating different types of cancer. Some important CAR-T-cell therapies include Abecma (idecabtagene vicleucel) for the treatment of relapsed or refractory multiple myeloma [41], Lisocabtagene maraleucel (Breyanzi) for the treatment of relapsed or refractory large B-cell lymphoma [42], Brexucabtagene autoleucel (Tecartus) for the treatment of relapsed or refractory mantle cell lymphoma [43], and Liso-cel (liso-cel) for the treatment of relapsed or refractory large B-cell lymphoma [44].

The FDA approval of CAR-T-cell therapy also expanded to other types of cancer beyond leukemia and lymphoma, and there have been many ongoing clinical trials, with some being completed, which are summarized in Table 1.

## 5. Current Limitations and Potential Strategies

Despite CAR-T-cell therapy’s promising success in cancer treatment, several limitations still need to be addressed. These limitations make CAR-T-cell therapy less effective for treating certain types of cancer. Several factors cause cancer cells to become resistant to this therapy, such as antigen escape, on-target/off-tumor effects, poor CAR-T-cell trafficking and tumor infiltration, immunosuppressive TME, and CAR-T-cell therapy-associated toxicities. Also, to treat cancer and prevent relapse, CAR-T cells have to persist and remain active for a longer period of time in our body. The main limiting factors for CAR-T-cell persistence include the stability of transgene expression, immune responses against CAR, and the adaptation of CAR-T cells inside the body [45]. There are different factors that contribute to severe toxicities associated with CAR-T-cell therapy, which include disease burden [46], high-dose chemotherapy regimen [47], high-dose CAR-T-cell infusion [48], and peak levels of serum cytokines and C-reactive protein [49]. 

**Figure 4:**
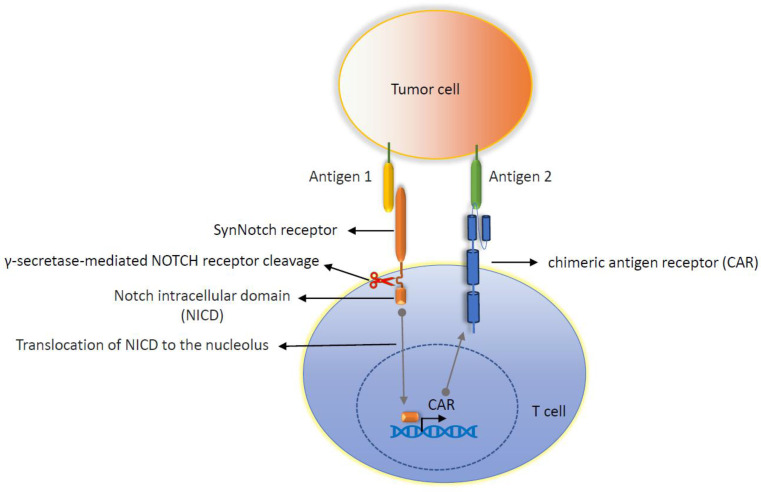
Cartoon diagram showing the strategy of targeting two antigens using an engineered synthetic Notch receptor-CAR circuit that drives the expression of CAR.

Most solid tumors create immunosuppressing TMEs that obstruct tumors’ infiltration, activation, or expansion of cytotoxic T cells. Many strategies have been proposed to overcome this limitation, including the local/systemic administration of high-dose inflammatory cytokines engineering T cells to express the synthetic Notch (synNotch) receptor-mediated local production of IL-2 [50,51,52,53]. Another limitation of CAR-T-cell therapy in solid tumor is the unavailability of tumor-specific antigens. Most of the antigens that have been targeted via CAR-T-cell therapy are tumor-associated, not tumor-specific, in nature, which means these antigens also express in low levels in normal tissues, leading to severe cytotoxicity. Some strategies showed effectiveness at overcoming this issue, which include targeting two antigens rather than one antigen using the synthetic Notch receptor and CAR, as shown in Figure 4 [54], engineered CAR-T cells with synthetic Notch receptors to sense antigen density [55], and targeting tumor-associated antigens that are post-translationally modified in tumors [56,57,58,59,60,61]. 

The response to different doses of CAR-T-cell therapy varies from patient to patient, so another challenge of this therapy is determining the correct dose for patients. Similarly, knowing whether a single dose or an infusion of multiple small doses has the optimum effect on patients is essential [36,62]. Likewise, ex vivo T-cell expansion duration is another confounding factor. In this regard, some studies showed that less differentiated and more proliferative T cells have better anti-tumor responses in preclinical trials [63,64]. There has been a massive amount of effort made to design innovative CAR-T-cell therapy strategies to overcome these limitations, which are elaborated in the subsequent paragraph and summarized in Table 2.

## 6. Emerging Combination Strategies with CAR-T-Cell Therapy

CAR-T-cell therapy, in combination with other therapies, revolutionized cancer treatment, especially for solid tumors. Many studies have shown that combination therapy with CAR-T-cell therapy dramatically improved the effectiveness and reduced the side effects of treatment [51,54,95,96]. This combination of approaches was found to modulate the tumor microenvironment, enhance the CAR-T structure, connect the CAR-T cells with cancer cells, increase precision by targeting multiple antigens at the same time, bypass the tumor immune escape, and reduce the side effects. Some important combination therapies are discussed in the sections below.

### 6.1. Combination of CAR-T-Cell Therapy with Chemotherapy

Past studies have found that chemotherapy can induce immune function and reduce tumor burden, highlighting the possible benefits of this therapy for improving CAR-T-cell function. As expected, a study noted that this combination approach upregulated the migration of DCs and T cells into the TME. Moreover, chemotherapy has been found to elevate the secretion of damage-associated molecular patterns (DAMPs) and high-mobility group box 1 (HMGB1) from apoptotic/necrotic tumor cells [97], which, consequently, may help with the CAR-T-cell function. Similarly, tumor cells that have experienced chemotherapy can produce type I interferon (IFN- I), which can also escalate the maturation of DCs [98]. 

Additionally, chemotherapy can suppress the function of different immunosuppressive cells, including regulatory T cells (Tregs) and myeloid-derived suppressor cells (MDSCs). Chemotherapy has been found to work synergistically with CAR-T cells for targeting extended ErbB family proteins in epithelial ovarian cancer (EOC) [99]. Similarly, a clinical trial identified the improved persistence and response of CD19 CAR-T cells in pediatric/young adult relapsed/refractory B-ALL after chemotherapy with cyclophosphamide (CTX) [100]. 

Moreover, chemotherapy reduces autoimmunity and the levels of immunosuppressive cells, and as a result, it induces the persistence of CAR-T cells [45,101]. Many chemotherapeutic agents, like cyclophosphamide, doxorubicin, and fluorouracil, have been shown to abrogate CAR-T-cell therapy’s function through various mechanisms [102,103]. Chemotherapy is found to sensitize the tumor cells by releasing granzyme B, which helps CAR-T cells to gain easy access to the antigens of cancer cells [97]. In comparison, CAR-T-cell therapy was also found to induce the efficiency of chemotherapy. A study reported that adaptive T-cells can reduce the resistance to chemotherapy by releasing the interferon-γ (IFN-γ) in ovarian cancer [104].

Chemotherapy-stimulated cancer-related macrophages are found to help with the attachment of CAR-T cells to the tumors. A study showed that the continuous synthesis of CCL5 by cancer cells attracts T-cells that release IFN-γ upon antigen identification [105]. Studies found that chemotherapeutic agents like taxanes and vinca alkaloids can induce calreticulin and create more tumor antigens, which subsequently help CAR-T cells in their activity [106]. Some clinical trials found an improvement in the event-free survival (EFS) time when patients received extensive lymphodepleting chemotherapy regimens [107,108].

### 6.2. Combination of CAR-T-Cell Therapy with Radiotherapy

Similar to chemotherapy, radiotherapy has been reported to modulate TME to promote CAR-T-cell infiltration and trafficking into tumor sites [109,110]. 

Radiotherapy can help cytotoxic lymphocytes to access tumor sites and kill cancer cells [111,112]. Additionally, it was noted that local radiotherapy might also help CAR-T cells to enter the TME by releasing different chemokines, including (CXCL) 1, 2, 9, 10, and 16, to help T-cells prepare for their entry into the TME [113].

Radiotherapy stimulates CTLs on the local site and provides an inhibitory effect against distant tumors [114]. Hence, radiotherapy can provide additional benefits via the suppression of metastasis. A past study showed a synergic effect of radiotherapy with natural killer group 2-member D (NKG2D)-based chimeric antigen receptor construct (chNKG2D) in fully immunocompetent orthotopic glioblastoma mouse models [115].

As for the monotherapy method, antigen escape can lead to the failure to treat solid tumors. A low dose of sensitizing radiation was shown to have a better treatment response for CAR-T-cell therapy via the mitigation of antigen escape [116].

### 6.3. Combination of CAR-T-Cell Therapy with Oncolytic Virus

Oncolytic virotherapy is a new, growing field in cancer immunotherapy, where killer viruses are selectively used to kill cancer cells. The most critical features of oncolytic viruses include a capacity to modify the genome of cancer cells, selective and direct treatment for cancer cells, and the recruitment of the innate and adaptive immune system to induce a tumor-specific immune response [117]. Oncolytic virotherapy can be used in various aspects, such as presenting tumor-associated antigens (TAA) to the immune system as a vaccine and delivering exogenous therapeutic genes to express inside the tumor; they can also be combined with other immunotherapy, including CAR-T-cell therapy [118]. Oncolytic viruses can synergize CAR-T-cell therapy to treat solid tumors for many reasons, including the diffusion of tumor antigens via the lytic effect on tumor cells, and carry potent therapeutic chemokines [119]. 

One of the main limitations of CAR-T-cell therapy in treating solid tumors is the low migration capacity of CAR-T cells to the TME; different studies have reported that combining CAR-T-cell therapy with an oncolytic virus improved the migration of CAR-T cells due to the enhanced M1 polarization of macrophages and the maturation of DCs [120,121,122]. Similarly, a study found improved CAR-T-cell migration when mesothelin targeted CAR-T-cell therapy combined with an oncolytic virus expressing TGF-β in a triple-negative breast cancer model [123]. Another study noted improved performance in HER2-targeted CAR-T cells by combining an oncolytic adenovirus expressing a programmed death-ligand 1 (PD-L1) blocking antibody [124]. Moreover, a report showed that when an oncolytic virus secreted IL-12p70, in addition to a PD-L1-blocking antibody combined with CAR-T-cell therapy in head and neck squamous cell carcinoma (HNSCC) xenograft models, it showed reduced growth primary and metastasized tumors compared to individual monotherapy [125]. In addition to improving the migration of CAR-T cells, this combination strategy was found to overcome antigen escape and the off-target effects of CAR-T cells [126]. In addition to the anti-tumor effects of CAR-T cells, this combination treatment was shown to induce tumor-specific immune memory.

### 6.4. Combination of CAR-T-Cell Therapy with Cancer Vaccines

Cancer vaccines are generally used to expose tumor-associated epitopes to activate the body’s adaptive immune system for eradicating cancer cells. Indeed, cancer vaccines have been found to enhance the function of CAR-T cells by inducing antigen-presenting cells (APCs) or human leukocyte antigen (HLA) expression and directly stimulating dual- or bispecific CAR-T-cells into the tumor site [127]. Based on the structural differences, cancer vaccines are divided into three types, as discussed in the sections below.

#### 6.4.1. Cellular Vaccines

In this type of cancer vaccine, whole cells/cellular components were used as sources of antigens for antigen-presenting cells. Cellular vaccines can be prepared from tumor cells, irradiated immortalized cell lines, or dendritic cells (DCs) [128]. This combination approach was first used in a whole-cell vaccine by engineering the K562 cell line to express the CMV-pp65 protein and the immune stimulatory molecules CD40 ligand CD40L and OX40 Ligand. Similarly, a clinical trial used an irradiated EBV-transformed lymphoblastoid cell line (LCL) as a cellular vaccine and identified better the persistence and expansion of CD19-CAR-CTL therapy [129].

DCs regulate innate and adaptive immune responses and present tumor antigens to T cells. To induce anti-tumor immune responses, DC-based vaccines were developed [128,130]. In CAR-T-cell therapy in blood cancer, tumor relapse is one of the limitations, so combining CAR-T-cell therapy with DC-based vaccines would be a good strategy. A group showed the better expansion and persistence of CAR-T cells via the Eps8-DC-mediated cellular vaccine [131].

#### 6.4.2. Molecular Vaccines

In molecular vaccines, different molecules have been used (including peptides, DNA, and RNA) to load specific antigens to the APCs and induce T cells. Thus, combining molecular vaccines with CAR-T-cell therapy would be beneficial for targeting specific antigens in tumor cells and inducing CAR-T-cell-medicated killing of cancer cells. Designing and using a specific vehicle is most important as this strategy requires delivering this molecular vaccine to the APC cells. A study used a nanoparticulate vaccination platform by encapsulating ovalbumin (OVA) peptides and found a durable remission in mice [132]. Similarly, another study used liposomal antigen-encoding RNA (RNA-LPX) and noted the significant expansion and cytolysis activity of claudin 6 (CLDN6) targeting CAR-T cells [133].

#### 6.4.3. Viral Vaccines

Many studies have investigated the use of complete viruses or virus antigens as immunogens to promote T-cell function [134,135,136]. A study restimulated CAR (+)-T cells through an endogenous cytomegalovirus (CMV)-specific T-cell receptor to enhance persistence and augment the anti-tumor activity of CD19-CAR-T cells [137]. In these bispecific T cells, both TCR and CAR are located on the same T cells, and they showed that vaccination with CMVpp65 abrogated the tumor eradication effects of CAR-T cells. Likewise, viral vaccine encoding human gp100 (VV-gp100) and showed enhanced expansion of Her2 CAR-T cells.

### 6.5. Combination of CAR-T-Cell Therapy with Cytokines

The TME is very immunosuppressive due to the production of various types of cytokines. To protect CAR-T cells from the immunosuppressive cytokine IL-4, a group created CAR-T cells with inverted cytokine receptors (ICRs) encoding the cytokine-binding portion of the IL-4 receptor (an immunosuppressive cytokine releases from TME) exodomain linked to the IL-7 receptor (an immunostimulatory cytokine) signaling endodomain. These CAR-T cells were directed against prostate stem cell antigen (PSCA) to target pancreatic cancer, and the authors found that these CAR-T cells persisted in an IL-4-rich TME, which resulted in enhanced anti-tumor activity [83]. Likewise, a group developed CAR-T cells with a novel 4/21 ICR, a fusion protein of the IL-4 receptor ectodomain and IL-21 receptor endodomain [138]. The whole purpose of this 4/21 ICR is to inhibit the immunosuppressive effects of IL-4 and activate STAT3 phosphorylation to ensure a better immune response [139]. Similarly, another group experimented with CAR-T cells expressing the IL-7 receptor (C7R) that targets AXL in AXL-positive triple-negative breast cancer (TNBC) [140]. This study noted that IL-7 expression augmented the cytotoxicity and survival of CAR-T cells in TNBC. 

IL-15 is another cytokine that stimulates the immune system by inducing T-cell persistence and trafficking [141]. In lymphoma and leukemia research, a study noted improved performance in CAR-T cells targeting CD19 via the expression of the IL-15 gene and an inducible caspase-9-based suicide gene [142]. In addition, this study noted that iC9/CAR.19/IL-15 cells reduced the expression of PD-1 receptors and blocked CAR-T-cell exhaustion. Similarly, another study investigated CAR-T/IL-15 cells in lymphoma and glioblastoma models, and they reported the downregulation of exhaustion markers and upregulation of antiapoptotic markers in CAR-T cells [143]. Likewise, combining murine IL-15 (mIL-5) with CAR-T cells was noted to upregulate Bcl-2 (an antiapoptotic marker) and decreased PD-1 expression [144]. For the multiple myeloma (MM) model, a group found IL15 to be superior when they compared different combinations of IL-15 and IL-2 cytokines in B-cell maturation antigen (BCMA)-targeted CAR-T cells [145]. For improving the persistence of CAR-T cells, T-memory stem cells (TSCM) play a significant role, so a group showed that membrane-bound chimeric IL-15 (mbIL-15) expression improved the persistence of the TSCM phenotype [59].

### 6.6. Combination of CAR-T-Cell Therapy with Checkpoint Inhibition

As tumor cells invade the immune system by expressing different immune checkpoint ligands, checkpoint blockade (CPB) via immune checkpoint inhibitors (CPIs) has become one of the most promising strategies for cancer treatment. Many studies investigated the combined effects of CAR-T cells with PD-1/PD-L1 CPB, which are summarized in the sections below.

#### 6.6.1. Antibodies-Mediated Checkpoint Blockade

Several studies have already reported that combining anti-PD-1 antibodies with CAR-T-cell therapy improved the persistence of CAR-T cells in different types of cancer [146]. A study showed enhanced therapeutic outcomes when they combined anti-Her-2 CD8 + T cells with anti-PD-1 antibodies in murine breast cancer models [147]. This study found that treatment with anti-PD-1 antibodies induced T-cell immunity by improving CAR-T cells’ activity and enhancing the expression of IFN-γ and granzyme B in both in vitro and in vivo conditions. Although this study was conducted in HER2-amplified cancer, it does not consider the possibility of an autoimmune effect on normal tissue expressing normal physiological levels of HER2 [147,148,149,150]. Despite a growing body of research showing that improved outcomes by combining the anti-PD-1 antibody therapy with CAR-T-cell therapy, there are still multiple concerns, including anti-PD-1 antibody barely reaching optimum persistence in TME when systemically infused. Also, this immune-augmentation strategy may cause uncontrolled T-cell activation; as a result, it has been found to affect several organs [151,152]. CPB antibodies can be delivered via CAR-T cells to overcome these cytotoxic effects. A study showed that preconditioning the CAR-T cells in the IL-7/IL-15 cytokines’ milieu could induce therapeutic outcomes and decrease treatment-related adverse events [153].

#### 6.6.2. DNR and shRNA-Mediated Checkpoint Blockade

PD-1 dominant negative receptor (DNR) can be expressed in CAR-T cells to reduce PD-1-mediated immunosuppression in TME [154]. In this approach, only the ECD of PD-L1 is expressed in CAR-T cells. PD-1 DNR reduces the engagement of endogenous PD 1 receptor with PD-L1 via competitive binding and reduces the immune suppression of cancer cells [154]. In this study, the authors also made CAR-T cells with PD-1–targeting shRNAs and showed the improved performance of CAR-T cells [154]. Retrovirus-mediated shRNA targeting adenosine 2A receptors (A2ARs) was examined in HER2-targeted CAR-T cells, and we found improved function in CAR-T cells [155]. Likewise, the knockdown of cytotoxic T-lymphocyte associated protein 4 (CTLA-4) in CAR-T cells via shRNA also showed beneficial effects for the performance of CAR-T cells [156].

#### 6.6.3. Checkpoint Blockade via Gene Editing

##### Single-Gene Editing

In 2019, a group used the CRISPR/Cas9 tool to delete the programmed cell death protein 1 (PDCD1) gene in CAR-T cells targeting mesothelin. This study found that knocking out the PDCD1 gene improved the CAR-T performance [157]. Likewise, another study showed that the CRISPR/Cas9-mediated deletion of the PDCD1 gene increased the cytotoxic effect of CD19 targeting CAR-T cells and reduced tumor growth in a xenograft tumor model [158]. Similarly, CRISPR/Cas9 mediated the deletion of lymphocyte activation gene-3 (LAG-3) (a negative regulator of T-cell activity) was found to improve the anti-tumor effect of CAR-T cells in the murine xenograft model [159].

##### Multiplex Gene Editing

Similar to single-gene editing, altering multiple genes via CRISPR/Cas9 seems to be a good strategy for making universal allogeneic T cells, improving the treatment efficacy, and reducing both time and expense of CAR-T-cell therapy. Graft-versus-host disease (GVHD) is a primary concern in using allogeneic T cells, so silencing endogenous TCR and β-2 microglobulin (an essential subunit of the HLA-I) may eliminate the limitation associated with GVHD. As a result, CRISPR/Cas9-mediated CBP therapy become a novel area of interest in cancer immunotherapy. Similarly, to improve the persistence of CAR-T cells, the deletion of CTLA-4 and PD1 was achieved, and the findings suggest that it also reduces the resistance to apoptosis and the immune inhibition of CAR-T cells [160]. 

##### Co-Stimulatory Molecule

Previous studies reported that adding co-stimulatory domains like 4-1BB and CD28 in CAR-T cells improved their performance. It was also noted that the selection and positioning of the co-stimulatory molecules in the CAR construct affect CAR-T cells’ function, kinetics, and potential safety profile. Studies found that compared to CD28, the incorporation of 4-1BB in CAR-T cells improved CAR-T cells performance via the significant downregulation of T cells’ immunoglobulin and mucin domain-containing protein 3 (TIM3), LAG-3, and PD-1 [154,161]. Different types of cancer may benefit from different CAR-T-cell co-stimulatory domains; for example, using the 4-1BB co-stimulatory domain for longer persistence may be necessary for the long-term remission of the precursor B-cell malignancy B-ALL, whereas it is less critical than early antitumor activity when treating mature B-NHL malignancies [162]. 

##### Pharmacological Antagonists

Checkpoint blockade via pharmacological means has also been tested via CAR-T-cell therapy. A study showed that combining A2AR antagonist SCH58261 with anti-PD-1 antibody improved CAR-T-cell function and upregulated granzyme B expression in tumor-infiltrating CD8 + T cells [155]. 

### 6.7. Combination of CAR-T-Cell Therapy with BiTEs

Bispecific T-cell engagers (BiTEs) have a dual specificity against two different antigens [163]. The benefits of this strategy are that it can activate T cells and link T cells with tumor cells without engaging MHC [163]. A group examined the efficacy of CD3/EGFR BiTEs together with EGFRvIII (oncogenic mutation in the ECD of the EGFR)-targeted CAR-T cells in a glioblastoma mouse model and showed that this strategy induced the efficiency of CAR-T cells function by removing heterogeneous tumor cells without significant toxicity [164,165].

As the loss of CD19 was the main limitation in using anti-CD19-CAR-T-cell therapy, to overcome this limitation, blinatumomab (a BiTE composed of a CD3 and a CD19 site) was combined with anti-CD19 CAR-T cells and tested in relapsed/refractory B-ALL cases, and the findings showed that blinatumomab augmented CAR-T performance [166]. In another study, when anti-biotin CAR-T cells were combined with biotinylated bispecific antibody (anti-CD19 or anti-CD20) coated on tumor cells, the function of CAR-T cells improved [167]. As the folate receptor (FR) is found to be overexpressed in tumors (including lung, ovarian, uterus, and breast tumors) compared to normal tissue, folate fluorescein isothiocyanate (FITC) conjugate (BiTE) was developed to control CAR-T cells’ function, as well as target FR [168]. Combining anti-FITC-CAR-T cells with BiTE (folate-FITC conjugate) was found to recruit the CAR-T cells in the tumor site and induce their function in tumors with high FR expression [168]. Using this strategy, folate-FITC can be conjugated with diverse anti-tumor antibodies and directed to specific cancers [169]. 

### 6.8. Combination of CAR-T-Cell Therapy with Immunomodulatory Agents

Different immunomodulatory drugs have been tested with CAR-T-cell therapy and showed that this combination strategy significantly improved CAR-T cells’ proliferation, persistence, and cytokine production in the TME. Some of the immune modulatory drugs combined with CAR-T-cell therapy have been described in the following sections. 

#### 6.8.1. Lenalidomide

Lenalidomide has been shown to induce the proliferation and cytokine production of T cells by inhibiting the immunosuppressive effect of CTLA4-Ig (a blocker of the B7-CD28 pathway) [170,171]. In these studies, lenalidomide was found to induce the expression of NF-κB and enhance the phosphorylation of the CD28 co-stimulatory molecule in T cells. Hence, combination treatment with lenalidomide and CAR-T-cell therapy will be an excellent strategy to overcome the limitations associated with the immunosuppressive environment of TME. Likewise, another study found that lenalidomide, in combination with CD19-CAR-T-cell therapy, induced IFN-γ production; as a result, it caused elevated activity and infiltration of T cells in TME and reduced tumor burden in mice [172]. Similarly, a study reported the lenalidomide-mediated increased persistence and cytotoxicity of CAR-T cells in multiple myeloma models [173]. This study noted that this combination strategy not only reinforced the immunological synapse formation between CAR-T cells and myeloma cells but also induced the proliferation of CTLs, enhanced the production of immunogenic cytokines like IFN-γ and TNF-α, and inhibited the production of immunosuppressive cytokines, like IL-5 and IL-10. 

#### 6.8.2. miRNAs (miR-153)

Similar to small-molecule drugs, non-coding RNAs also emerge as epigenetic immunomodulatory molecules [174,175,176,177,178], which are reported to affect naive, effector, and memory T-cell function. miR-153 was found to inhibit the expression of Indol amine 2,3-dioxygenase 1 (IDO1), which catalyzes the production of tryptophan kynurenine and 3-hydroxy anthranilic acid (immunosuppressive metabolites). A study reported that the overexpression of miR-153 with anti-EGFRvIII-CAR-T cells therapy reduced tumor burden in the xenografts model of colon cancer [179]. As IDO catalyzes the conversion of tryptophan into inhibitory metabolites that inhibit T-cell activity, IDO inhibitors were noted to enhance the efficacy of CD19-CAR-T-cell therapy in a xenograft lymphoma model [180].

#### 6.8.3. Decitabine

Antigen heterogeneity and the loss of antigen is one of the main limitations of CAR-T-cell therapy [181,182]. Decitabine, a hypomethylating compound, was found to augment the effect of CAR-T cells. A study showed that decitabine increased the expression of mucin 1 (MUC1) by demethylating DNA in pancreatic tumor cells, and decitabine was found to increase the potency of MUC1-CAR-T cells. Another study reported that decitabine improved the efficacy of CD19-CAR-T-cell therapy by upregulating the expression level of CD19 in lymphoma cell lines [183]. Similarly, on acute myeloid leukemia (AML) cell lines, decitabine was found to improve the effectiveness of CD33-CAR-T cells by enhancing the expression of CD33 [184].

#### 6.8.4. HDAC Inhibitors (HDACis)

The acetylation of histone in chromatin was found to open a compact chromatin structure, and so it helps with transcription factor binding for gene expression process (transcription). Similarly, histone deacetylase (HDAC) can deacetylate histones and suppress the transcription of related genes [185]; hence, utilizing HDAC inhibitors (HDACis) seems to be an excellent strategy for inducing specific gene expression [186]. Studies reported that when Romidepsin (HDAC inhibitors) is combined with CD20-CAR-T-cell therapy, it induces the expression of CD20, elevates the potency of CD20-CAR-T cells, and prolongs the overall survival of the mice in the lymphoma model [187]. Similarly, another study showed that administration of HDACi valproic acid before CAR-T-cell therapy induced the expression levels of NKG2DL on low-level expressing AML cell lines, and as a result, it enhanced the anti-tumor effects of CAR-T cells [188].

#### 6.8.5. SMAC Mimetics

The second mitochondria-derived activator of caspase (SMAC) mimetics binds to inhibitor of apoptosis proteins (IAPs), thus freeing caspases to activate apoptosis [189]. Inhibition of IAPs via SMAC mimetics (SMs) seems to be a good strategy for improving the CAR-T cell’s function. A study found three SMs to be effective boosters of CD19-CAR-T cells in B-ALL [190]. Similarly, another study showed that administering birinapant (a potent SM) with HER2-CAR-T cells improved its efficacy by engaging the TNF receptor pathway; as a result, it reduced tumor growth in the murine model [191].

### 6.9. Combination of CAR-T-Cell Therapy with Allo-HSCT

Allogeneic hematopoietic stem cell transplantation (allo-HSCT) is a treatment strategy of adoptive cell therapy (ACT), and it can be combined with CAR-T-cell therapy for cancer treatment [192]. Studies have shown that using allo-HSCT before CAR-T-cell therapy improved the effectiveness of CAR-T cells in relapsed/refractory (R/R) B-ALL patients with minimal residual disease (MRD) [193,194]. This combination therapy with CD19-CAR-T cells and allo-HSCT was shown to prolong leukemia-free survival (LFS) with acceptable safety and efficacy compared to CAR-T-cell monotherapy [195,196]. Similarly, augmented effects in tumor eradication with prolonged EFS (event free survival) and OS (overall survival) were shown in combination therapy with CD22 CAR-T-cell therapy and allo-HSCT [197,198]. Likewise, allo-HSCT after CD19-targeted CAR-T-cell therapy was found to be safe and effective at treating B-cell chronic lymphocytic leukemia (B-CLL) and non-Hodgkin lymphoma (NHL) [199].

### 6.10. Combination of CAR-T-Cell Therapy with Metabolic Inhibitors

In particular, for solid tumors, manipulating the metabolic profile of TME to induce the effectiveness and persistence of CAR-T-cell therapy is a promising strategy [200]. Different genetic modifications have been made in T cells to modify the metabolic profile of TME, such as upregulating mitochondrial function via the ectopic expression of PCG1α (PPAR-γ co-activator 1α), inducing effector T-cell functions via the knocking out of ACAT1 (acyl-CoA cholesterol acyltransferase 1), reducing hypoxia by upregulating the expression of catalase, etc. [201,202,203,204,205,206,207]. In addition to these, different co-stimulatory molecules have been added to CAR-T cells to enhance the survival, proliferation, and effectiveness of CAR-T cells in different TMEs, which include adding CD28 for aerobic glycolysis, adding 4-1BB for fatty acid oxidative breakdown and mitochondrial biogenesis, etc. [161,208,209,210,211]. The presence of immunosuppressive cytokines like PGE2, IDO, CTLA4, PD-L1, IL-10, and TGF-β released in TME can affect the efficacy of CAR-T-cell therapy [212]. A study showed that the treatment with all-trans retinoic acid (ATRA) improved the efficacy of CAR-T cells by reducing the suppressive effects of myeloid-derived suppressor cells (MDSCs) in pediatric sarcomas [213]. Similarly, another group found that the expression of NKG2D ligands via NK cells significantly abolished MDSCs and augmented the anti-tumor tumor effects of CAR-T cells [214]. In the same way, to inhibit the immunosuppressive effects of IDO, IDO inhibitory drugs such as cyclophosphamide and fludarabine have been used before CAR-T-cell therapy, reporting significant tumor regression and increased survival [180]. 

## 7. Conclusions

So far, we have outlined the considerable efforts of researchers in the field of CAR-T cells’ evolution, and it is an integral part of the armamentarium in the field of hematological malignancies. Although this treatment can be indicated for the treatment of both hematological malignancies and solid tumors, a game-changing role for these agents has clearly emerged in treating hematological malignancies. In contrast, the promises in the field of solid tumors have yet to be fulfilled due to different limitations in the treatment of solid tumors. Lack of specific TAA is a major limitation for solid tumors, contributing to antigen escape, on-target/off-tumor effects, and life-threatening adverse effects such as cytokine storm syndrome and tumor lysis syndrome. It has been noted that major regulatory agencies like the FDA in the US and the European Medicines Agency (EMA) in Europe have approved a large number of CAR-T therapies for clinical use in hematological malignancies. After many years of research into CAR-T therapies, the technical modifications of this therapy have already been achieved in treating solid tumors. These modifications include targeting multiple targets, immune checkpoint blockade, etc. Many combination strategies with existing treatments were also applied, which showed promising results for improving the efficacy of CAR-T therapy in both solid and hematological malignancies [215,216]. Indeed, more research and advanced knowledge in CAR-T-cell therapy are necessary to treat solid tumors in the near future.

## Figures and Tables

**Table 1:** Important clinical trials for CAR-T-cell therapy in cancer that are currently ongoing or completed.

NCT Number	CAR-T Strategy	Conditions	Type of Tumor	Phase	Current Status	Enrollment	Estimated/Actual Completion Date (DD Month YYYY)	Sponsor
NCT02348216	CD19-specific CAR-T cells	Diffuse large B-cell lymphoma (DLBCL), primary mediastinal large B-cell lymphoma (PMBCL), transformation follicular lymphoma (TFL), high-grade B-cell lymphoma (HGBCL)	Hematological malignancy	Phase 1 Phase 2	Completed	307	27 July 2023	Kite, A Gilead Company, Santa Monica, CA, USA
NCT02445248	CD19-specific CAR-T cells	Relapsed or refractory DLBCL		Phase 2	Completed	115	22 December 2022	Novartis Pharmaceuticals, Basel Switzerland
NCT02601313	CD19-specific CAR-T cells	Relapsed/refractory mantle cell lymphoma		Phase 2	Active, not recruiting	105	July 2025	Kite, A Gilead Company
NCT02614066	CD19-specific CAR-T cells	Relapsed/refractory B-precursor acute lymphoblastic leukemia		Phase 1 Phase 2	Active, not recruiting	125	November 2034	Kite, A Gilead Company
NCT02631044	CD19-specific CAR-T cells	Non-Hodgkin lymphoma, diffuse large B cell lymphoma, follicular lymphoma, mantle-cell lymphoma, primary mediastinal B-cell lymphoma		Phase 1	Active, not recruiting	385	10 May 2024	Juno Therapeutics, a Subsidiary of Celgene, Seattle, WA, USA
NCT02926833	CD19-specific CAR-T cells in combination with PD-1 antibodies	DLBCL		Phase 1 Phase 2	Completed	37	12 January 2023	Kite, A Gilead Company
NCT03105336	CD19-specific CAR-T cells	Refractory/relapse large B cell lymphoma		Phase 2	Active, not recruiting	159	Spetember 2036	Kite, A Gilead Company
NCT03287817	CD19- and CD22-specific CAR-T cells, followed by anti-PD1 antibody	DLBCL		Phase 1 Phase 2	Active, not recruiting	73	November 2024	Autolus Limited, London, UK
NCT03310619	CD19-specific CAR-T cells	Aggressive B-NHL		Phase 1 Phase 2	Completed	62	15 February 2023	Celgene, Summit, NJ, USA
NCT03568461	CD19-specific CAR-T cells	Refractory follicular lymphoma		Phase 2	Active, not recruiting	98	22 May 2025	Novartis Pharmaceuticals
NCT00004178	CEA CAR	Adenocarcinoma	Solid tumor	Phase 1	Completed	Not given	December 2001	Roger Williams Medical Center, Providence, RI, USA
NCT00019136	Folate receptor CAR ± IL-2	Ovarian cancer		Phase 1	Completed	Not given	Not given	National Cancer Institute (NCI), Rockville, MD, USA
NCT00085930	GD2 CAR, EBV T cells	Neuroblastoma		Phase 1	Active, not recruiting	19	December 2023	Baylor College of Medicine, Houston, TX, USA
NCT00730613	IL-13Ra2 targeting CAR-T cells	Glioblastoma with Hy/TK suicide switch		Phase 1	Completed	3	August 2011	City of Hope Medical Center, Duarte, CA, USA
NCT00889954	HER2 CAR, EBV T cells + TGFb DNR	HER2-positive lung cancer		Phase 1	Completed	20	21 January 2018	Baylor College of Medicine
NCT00902044	HER2 CD28 CAR	HER2-positive sarcoma		Phase 1	Active, not recruiting	36	July 2032	Baylor College of Medicine
NCT01109095	Her2 CAR, CMV T cells	HER2-positive glioblastoma		Phase 1	Completed	16	7 March 2018	Baylor College of Medicine
NCT01140373	PSMA CAR 2nd	Castrate metastatic		Phase 1	Active, not recruiting	13	June 2024	Memorial Sloan Kettering Cancer Center, New York, NY, USA
NCT01373047	CEA CAR	CEA-positive liver metastases		Phase 1	Completed	8	July 2013	Roger Williams Medical Center
NCT01454596	EGFRvIII CAR 3rd 28 and 4-1BB ± IL-2	Glioblastoma		Phase 1 Phase 2	Completed	18	17 January 2019	National Cancer Institute (NCI)
NCT01460901	GD2 CAR multivirus specific	Post-allo HSCT neuroblastoma		Phase 1	Completed	5	January 2015	Children’s Mercy Hospital Kansas City, Kanzas City, MO, USA
NCT01822652	GD-2-CAR-T with iCaspase9 Suicide safety switch	Neuroblastoma		Phase 1	Active, not recruiting	11	October 2030	Baylor College of Medicine
NCT02414269	Meso-CART cells, modified with iCasp9/M284	Malignant pleural disease		Phase 1 Phase 2	Active, not recruiting	113	30 April 2024	Memorial Sloan Kettering Cancer Center
NCT03170141	EGFRvIlI-specific CAR-T cells producing PD-1 and PD-L1 antibodies	Glioblastoma multiforme		Phase 1	Enrolling by invitation	20	31 Decemebr 2023	Shenzhen Geno-Immune Medical Institute, Shenzhen, China

**Table 2:** Current limitations and potential strategies of CAR-T-cell therapy.

Limitations	Potential Strategy	Supporting Reports	References
Antigen escape	Targeting multiple antigens	In preclinical trails, CAR-T cells targeting both CD19 and CD22 antigens in ALL/DLBCL and CAR-T cells targeting both CD19 and BCMA antigens in multiple myeloma have shown promising results.	[65,66,67,68]
		Similarly, in solid tumors, CAR-T cells targeting both HER2 and IL13Ra2 antigens in glioblastoma and CAR-T cells targeting both HER2 and MUC1 in breast cancer showed better antitumor effects compared to targeting a single antigen.	[56,69]
On-target/off-tumor effects	Targeting tumor-associated antigens that are post-translationally modified and only express in tumors	Different post-translationally modified tumor associated antigens such as TAG72, B7-H3, MUC1, and MUC16 have been targeted using CAR-T-cell therapy which showed very effective.	[56,57,58,59,60,61]
	Targeting two antigens rather than one antigen	Engineered CAR-T cells with synthetic Notch receptors to activate CAR targeting of the second antigen in the presence of the first antigen.	[54,70]
	Engineered CAR-T cells to sense high antigen densities in tumors	Engineered CAR-T cells with synthetic Notch receptors to activate CAR in a high antigen threshold.	[55]
Poor CAR-T-cell trafficking and tumor infiltration	Local administration vs. systemic delivery	Better antitumor effects were noted via the local delivery of CAR-T cells in glioblastoma cancer patients and the systemic delivery of CAR-T cells in mesothelioma patients.	[38,71,72]
	Expressing chemokine receptors on CAR-T cells that respond to tumor-derived chemokines	CAR-T cells that express CXCR1/CXCR2 enhanced trafficking and significantly improved antitumor efficacy.	[73,74,75]
	Genetically modified CAR-T cells that express proteins that help in the penetration of tumor stoma	CAR-T cells that express heparanase or CAR-T cells that target fibroblast activation protein have shown enhanced infiltration and antitumor activity.	[76,77]
Immunosuppressive TME	Combination checkpoint blockade with CAR-T-cell therapy	Administration of PD-1 inhibitor with CD19 CAR-T-cell therapy showed improved CAR-T-cell persistence in B-ALL patients.	[78]
		Likewise, in solid tumors, checkpoint blockade has been combined with CAR-T therapy and shown improved persistence of CAR-T cells.	[79,80]
	Engineering CAR-T cells to provide immunostimulatory signals to the TME	Better response was observed when CAR-T cells were genetically engineered to express immunostimulatory molecules like IL-12 and IL-15 and redirect immunosuppressive molecules like IL-4	[81,82,83]
		Genetic alteration of CAR-T cells to make them resistant to immunosuppressive factors like TGF β.	[84]
CAR-T-cell therapy associated toxicities	Altering CAR structure to ameliorate toxicity	Decreasing CAR antigen-binding domain affinity to micromolar affinity.	[85]
		Modulation of cytokine secretion via modifying the CAR hinge and transmembrane regions.	[86]
		Tailoring the costimulatory domain of CAR based on tumor type, tumor burden, antigen density, etc.	[87]
		CAR-mediated immune response can be decreased using human/humanized antibody fragments instead of murine-derived CARs.	[88,89]
	Modifying CAR transduced T cells and neurotoxicity	Inhibition of macrophage-activating and monocyte-activating cytokine GM-CSF with lenzilumab decreases cytokine-release syndrome and neurotoxicity.	[90,91]
		Administration of IL-1 receptor antagonists reduced a form of neuroinflammation in leukemia/lymphoma mouse models.	[92]
	CAR “off-switches”	CAR constructs engineered to express CD20 that helped with the depletion of CAR-T cells via rituximab treatment.	[93]
		Dasatinib treatment has exciting potential, as it provides the temporary inhibition of CAR-T-cell function.	[94]

## Data Availability

No new data were created for this study.

## Abbreviations

CAR	Chimeric antigen receptor
TME	Tumor microenvironment
ACT	Adoptive T-cell transfer
MHC	Major histocompatibility complex
TAA	Tumor-associated antigen
FasL	Fas ligand
TRAIL	Tumor necrosis factor-related apoptosis-inducing ligand
FDA	The Food and Drug Administration
EMA	The European Medicines Agency
EGFR	Epidermal growth factor receptor
HER2	Human epidermal growth factor receptor 2
HNSCC	Head and neck squamous cell carcinoma
CEA	Carcinoembryonic antigen
PSMA	Prostate-specific membrane antigen
scFv	Single-chain variable fragment
CD	Cluster of differentiation
NSCLC	Non-small-cell lung cancers
OS	Overall survival
PD-1	Programmed cell death-1
PFS	Progression-free survival
CSD	Co-stimulatory domain
IL	Interleukin
CRISPR	Clustered regularly interspaced short palindromic repeats
TCR	T-cell receptor
CLL	Chronic lymphocytic leukemia
ALL	Acute lymphoblastic leukemia
DLBCL	Diffuse large B-cell lymphoma
TNBC	Triple-negative breast cancer
TLR4	Toll-like receptor 4
DCs	Dendritic cells
DAMPs	Damage-associated molecular patterns
HMGB1	High-mobility group box 1
IFN-I	Type I interferon
Tregs	Regulatory T cells
MDSC	Myeloid-derived suppressor cells
TGF-β	Transforming growth factor-Β
EOC	Epithelial ovarian cancer
CTX	Cyclophosphamide
M6P	Mannose-6-phosphate
HLA	Human leukocyte antigen
APCs	Antigen-presenting cells
BCMA	B-Cell maturation antigen
CPIs	Checkpoint inhibitors
DNR	PD-1 dominant negative receptor
shRNA	Short hairpin RNA
A2ARs	Adenosine 2A receptors
CTLA-4	Cytotoxic T-lymphocyte associated protein 4
PDCD1	Programmed cell death protein 1
TRAC	T-Cell receptor alpha constant
BiTEs	Bispecific T-Cell engager
HDAC	Histone deacetylase
